# Innovative Applications of Artificial Intelligence in Bacteriophage Research: A New Chapter in Future Medicine

**DOI:** 10.3390/microorganisms14092013

**Published:** 2026-09-10

**Authors:** Dapeng Yang, Xin Yuan, Yubao Li

**Affiliations:** 1 College of Agriculture and Biology, Liaocheng University, Liaocheng 252000, China; ydp0503@163.com; 2 School of Traditional Chinese Pharmacy, China Pharmaceutical University, Nanjing 210000, China; 18418409608@163.com

**Keywords:** phage, artificial intelligence, machine learning, deep learning models, phage-host interaction

## Abstract

As the crisis of antibiotic resistance escalates, phage therapy has regained attention as an alternative strategy. Artificial intelligence (AI) technologies offer new avenues to overcome the bottlenecks inherent in traditional bacteriophage research. This review summarizes the multi-dimensional innovative applications of machine learning, deep learning, and large biological models in phage studies. In the fields of phage recognition and genomics, support vector machines (SVMs), convolutional neural networks (CNNs), and pre-trained protein language models can all achieve recognition accuracy rates of over 90%. Furthermore, tools such as DeepHost and VirSorter2 can efficiently identify phage sequences, annotate functional genes, and predict hosts at the species or strain levels. For clinical translation, AI integrates patient characteristics, bacterial phenotypes, and phage profiles to customize cocktail regimens for individualized phage therapy. Graph neural network-based models like DeepPBI-KG integrate multi-omics knowledge graphs to precisely predict phage-host interactions (PHIs), whereas agent-based simulation and defense protein predictors forecast phage resistance evolution. Additionally, generative AI can support the de novo design of functional phage genomes and mine massive unannotated virome dark matter. Nevertheless, this cross-disciplinary field faces significant constraints, including uneven and biased sequencing datasets, insufficient model interpretability, and dual-use biosafety ethical risks accompanied by unclear algorithm accountability and incomplete global supervision systems. Future research should optimize standardized multimodal databases, develop explainable AI algorithms, and establish cross-disciplinary ethical governance frameworks to facilitate closed-loop verification between computational prediction and wet-lab experiments. In conclusion, the deep integration of AI and phage biology provides revolutionary strategies to tackle multidrug-resistant infections and advances the clinical transformation of phage precision medicine.

## 1. Introduction

### 1.1. Research Background

With the escalating crisis of antibiotic resistance, the global public health system is facing unprecedented challenges. The World Health Organization has identified antibiotic resistance as one of the top ten threats to human health and has projected that it could cause 10 million deaths annually by 2050 [1,2]. Against this backdrop, bacteriophage therapy has re-entered the scientific spotlight as a potential alternative to antibiotics. Phage therapy has evolved as an important tool in precision medicine because of its ability to specifically target and eliminate bacterial pathogens without disrupting the human microbiota [3,4,5]. However, traditional approaches to phage research face multiple bottlenecks, including low screening efficiency, difficulty in predicting host range, and complex mechanisms underlying phage-host interaction (PHI), all of which severely constrain the clinical translation of phage therapy.

In recent years, the rapid advancement of AI technologies has opened new possibilities for overcoming these bottlenecks. Machine learning algorithms can identify patterns imperceptible to humans from massive genomic datasets, whereas deep learning models enable accurate prediction of protein structures and interactions [6,7]. Furthermore, natural language processing techniques are accelerating knowledge mining and synthesis from scientific literature. The integration of these powerful computational tools with bacteriophage research is profoundly transforming the research paradigm.

AI has enabled multidimensional innovative applications in phage research. In the field of phage identification, machine learning and deep learning models have achieved efficient and accurate phage identification and host prediction. For example, the support vector machine model achieves an identification accuracy exceeding 90%. In genomic research, AI tools facilitate phage sequence recognition, functional gene decoding, and metagenomic assembly, significantly improving the efficiency of genomic analysis. With respect to the advancement of phage therapy, AI promotes customization of personalized treatment regimens and enables precise prediction of PHI. For example, the DeepPBI-KG model, which integrates multidimensional information to enhance predictive performance. Furthermore, AI plays a key role in simulating the dynamic interplay between phages and bacteria, predicting the development of phage resistance, and comprehensively mining phage omics data.

Nevertheless, the application of AI in phage research also faces challenges, including data bias, ethical risks, and insufficient model interpretability. This article reviews the innovative applications of AI technologies in bacteriophage research, explores how they can accelerate the translation of phage therapy from the laboratory to the clinic, and examines the ethical challenges and technical limitations in this interdisciplinary field. By proposing strategies to improve data systems, strengthen ethical oversight, and enhance model performance, this review aims to provide new perspectives and ideas for addressing the global antibiotic resistance crisis and advancing the development of medicine.

### 1.2. Literature Search and Screening Strategy

Literature screening and retrieval in this review were systematically performed following standard narrative review procedures. Core biomedical and interdisciplinary databases including PubMed, Web of Science Core Collection, Scopus, and Google Scholar were searched to cover studies regarding artificial intelligence and phage research. The search strategy was constructed based on Boolean operators, and the main search keywords included: “bacteriophage”, “phage therapy”, “antibiotic resistance”, “artificial intelligence”, “machine learning”, “deep learning”, “predictive model”, and “model interpretability”. Synonymous terms and variant expressions were appropriately supplemented to maximize literature coverage and avoid missing relevant studies.

The inclusion criteria were set as follows: (1) peer-reviewed original research articles and review papers focusing on AI applications in phage research, phage therapy optimization, and antibiotic resistance mitigation; (2) studies published in English with accessible full-text content; (3) literature published from 2015 to 2026 to ensure the timeliness and novelty of interdisciplinary research progress, while classic landmark studies published earlier were selectively retained. The exclusion criteria included: (1) letters, conference abstracts, editorial comments, and non-peer-reviewed grey literature without complete experimental or analytical data; (2) studies merely focusing on artificial intelligence algorithms or phage basic biology without combining the two research fields; (3) repetitive publications, low-quality studies with incomplete data and ambiguous conclusions, and literature irrelevant to the clinical translation of phage therapy and AI technical limitations. All retrieved literature was first screened by title and abstract, followed by full-text evaluation to confirm final inclusion, ensuring the accuracy and reliability of the literature basis of this review.

## 2. Application of AI in Bacteriophage Recognition

**Figure 1:**
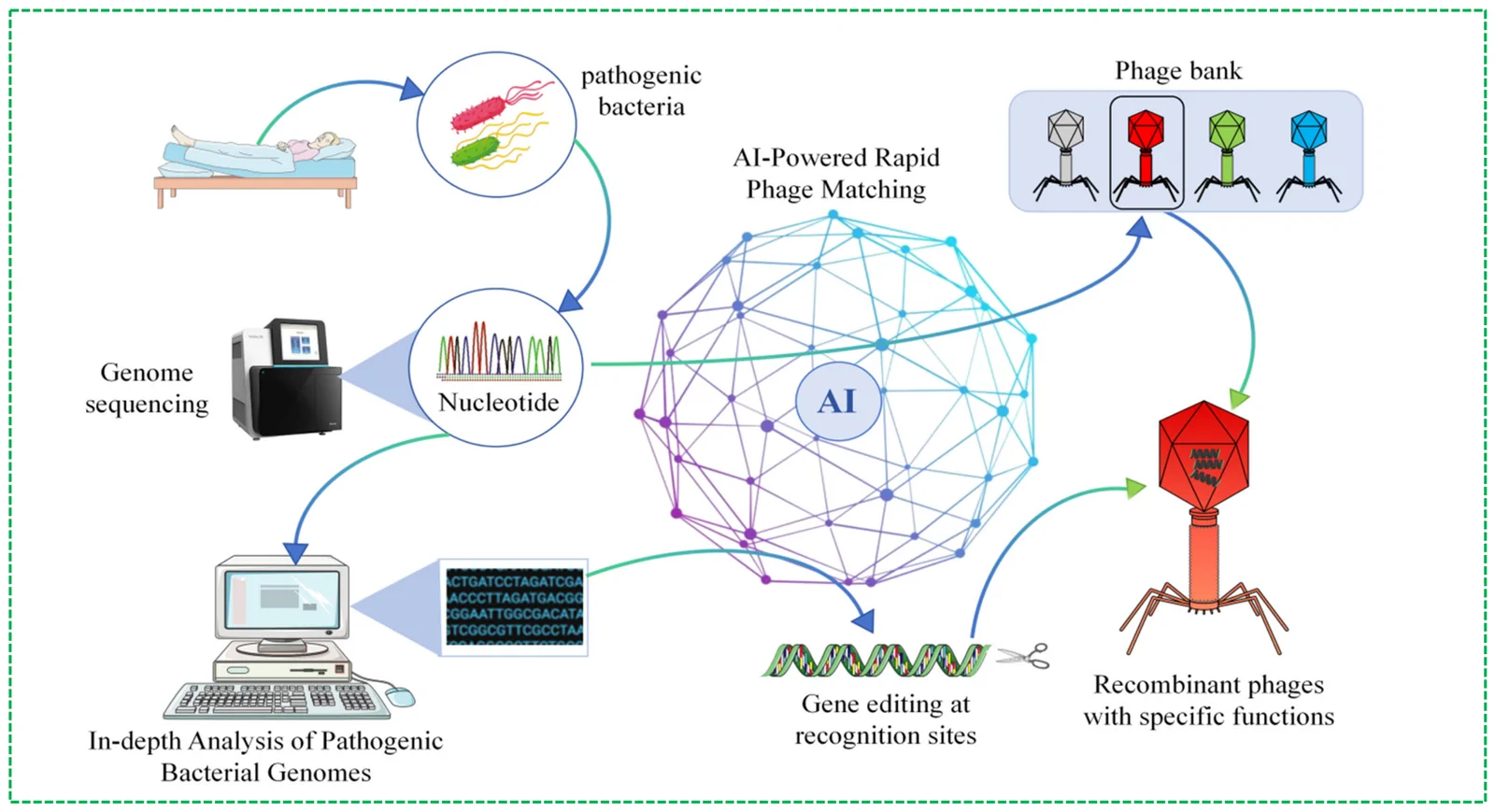
Rapid phage matching model for pathogenic bacteria. By using AI algorithms, the genomic data of pathogenic bacteria is analyzed to quickly match specific phages. Through AI analysis of the characteristics of pathogenic bacteria, highly efficient lytic recombinant phages are synthesized based on the characteristics of the pathogenic bacteria.

The introduction of AI technology has led to new breakthroughs in phage identification [8]. Phage recognition refers to the process of quickly distinguishing phage sequences from sequences of host bacteria and other species from environmental samples and sequencing data, and confirming the existence of phages [9]. The traditional recognition methods mainly rely on plaque culture observation and verification by PCR amplification. This process is cumbersome and time-consuming. Moreover, it is difficult to identify phages that cannot be cultured in vitro, and the missed detection rate is high [10]. Taking machine learning algorithms as an example, researchers have constructed analytical models capable of accurately identifying phages by training on feature data from many known phages [11]. Such models can rapidly screen raw high-throughput sequencing data directly, improving identification efficiency, effectively detecting uncultivable phages, and reducing the rate of missed detections. With the emergence of deep learning technology, phage recognition performance has been further improved. Compared with traditional machine learning methods, the deep learning model can automatically extract features from the original sequence data without manually designing empirical features, which reduces the workload of feature engineering. The recognition tool, based on convolutional neural networks, recurrent neural networks, and pre-trained large language models, can more accurately capture the potential characteristics of phage genomes. It can still stably and accurately distinguish phage sequences from host bacterial sequences despite the presence of mixed sequences in complex metagenomic sequencing data. It has obvious advantages in recognizing low-abundance phages and provides efficient technical support for mining new phages from environmental and clinical samples. Deep learning techniques have also demonstrated strong capabilities in phage identification. Deep learning models such as convolutional neural networks (CNNs) can automatically extract features from complex data like phage images and sequences [12,13]. In phage identification, CNNs can rapidly detect genetic information in phage sequences from metagenomic data [14,15,16]. The phage metagenome is the main source of unknown phage sequences in phage research. Traditional identification methods rely on homologous sequence alignment, which is inefficient for new phage sequences with low homology. The CNN model can quickly and accurately identify phage sequences from massive metagenomic data without relying on homologous alignment. This significantly improves the mining efficiency of unknown phages. The phage-host prediction tool called DeepHost has been developed, which uses a genome-encoding CNN to predict host taxonomy. DeepHost achieved 96.05% prediction accuracy at the genus level (72 taxa) and 90.78% at the species level (118 taxa) on its independent test set, outperforming previously published phage-host prediction tools by 10.16–30.48% [17]. Furthermore, the application of AI technology in phage identification extends to the rapid matching of phages against pathogenic bacteria. By analyzing phage gene expression data and combining it with AI algorithms, it is possible to predict the infectivity and lytic activity of phages against specific pathogenic bacteria, ultimately enabling the design of recombinant phages with tailored functions through synthesis (Figure 1). In phage research, AI technology serves as an advanced measurement tool, providing powerful means for accurate phage identification and understanding, thereby driving future medical applications of phages forward. The following table lists the currently most popular AI phage recognition tools and compares and analyzes their advantages and disadvantages (Table 1).

## 3. The Combination of Phage Genomics and AI

**Figure 2:**
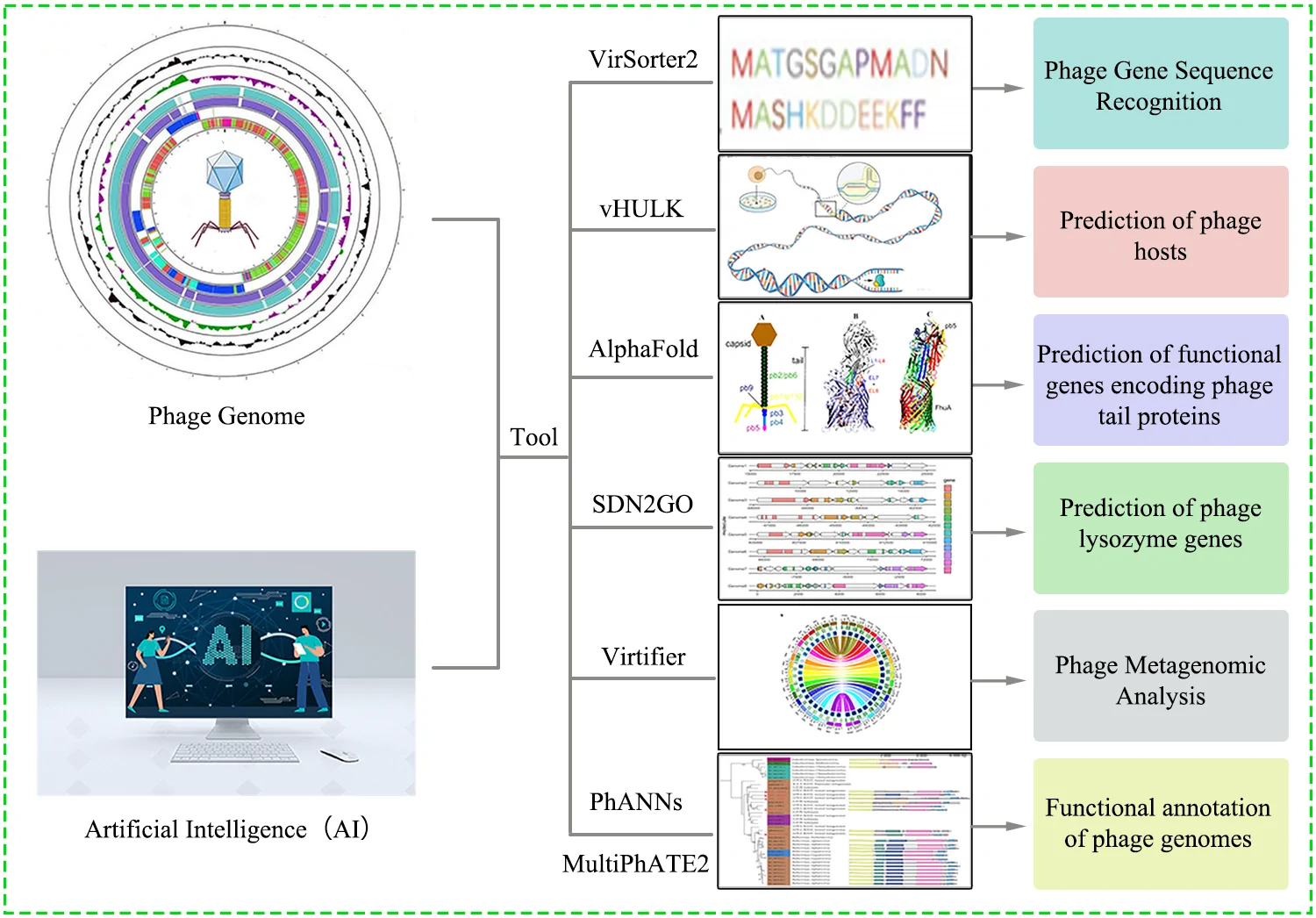
Application of AI in Phage Genomics Research.

Phage genomes carry genetic information that determines phage characteristics, including host specificity and infectivity [22]. AI has emerged as a powerful tool for deep mining of phage genomics data. Here we summarize several AI tools used in phage genomics research (Figure 2). First, during phage sequence identification and annotation, AI models effectively distinguish phage genomes from bacterial genome sequences and even identify chromosomal prophages. For instance, VirSorter2, with its ensemble learning strategy, significantly improves identification accuracy and sensitivity to novel phages [20]. DeepVirFinder uses a CNN to directly learn k-mer features and performs well in viral genome sequence identification [19]. Second, for taxonomic and host prediction, AI addresses the inefficiency of traditional methods when faced with large numbers of unknown phages. Using deep learning models, the vHULK tool achieves high-precision host prediction based solely on sequence data [23]. By combining random forests and neural networks, the RaFAH Framework integrates sequence data and CRISPR systems to enable robust host prediction [24,25]. The development of these AI tools offers strong technical support for phage genomics research and substantially improves the efficiency of phage genome analysis.

AI also serves as a powerful tool for decoding functional genes in phages. Despite the highly modular organization of phage genomes and the low rate of functional gene annotation, deep learning models based on protein language models and attention mechanisms can accurately predict genes encoding key functional proteins such as phage tail fiber proteins, lytic enzymes, and integrases [26,27]. AI models outperform traditional homology-based methods by a substantial margin in identifying receptor-binding proteins [28]. Concretely, deep learning architectures like CNNs and recurrent neural networks (RNNs) can automatically extract feature patterns related to receptor-binding proteins from vast stretches of phage genome sequences—patterns that conventional approaches often fail to capture [15,29]. Furthermore, specialized AI tools such as PhANNs and MultiPhATE2 are highly effective at identifying and assigning functions to phage genes [30,31]. At the level of structural genomics, protein structure prediction tools like AlphaFold have reshaped the research paradigm for key functional proteins, including phage tail fibers and lysozymes [32,33,34]. Such structural information helps elucidate the molecular mechanisms underlying phage infection of host bacteria and lays the groundwork for designing engineered phages.

Finally, AI is driving advancements in metagenomic assembly and ecological interpretation. Models such as Vibrant and VirFinder can be used for identifying sequences as well as assessing sequence completeness [18,35]. More importantly, graph neural networks and related models can integrate sequence data, abundance information, and environmental metadata to reveal the complex networks of PHI and their dynamics within ecosystems [36]. Furthermore, in metagenomic analyses, Virtifier outperforms several widely used methods in accurately identifying short viral sequences (<500 bp) [37]. These developments are shifting viral metagenomics research from isolated sequence analysis toward a more systematic understanding of ecology and evolution.

## 4. Utilizing Machine Learning to Optimize the Screening Process of Bacteriophages

**Figure 3:**
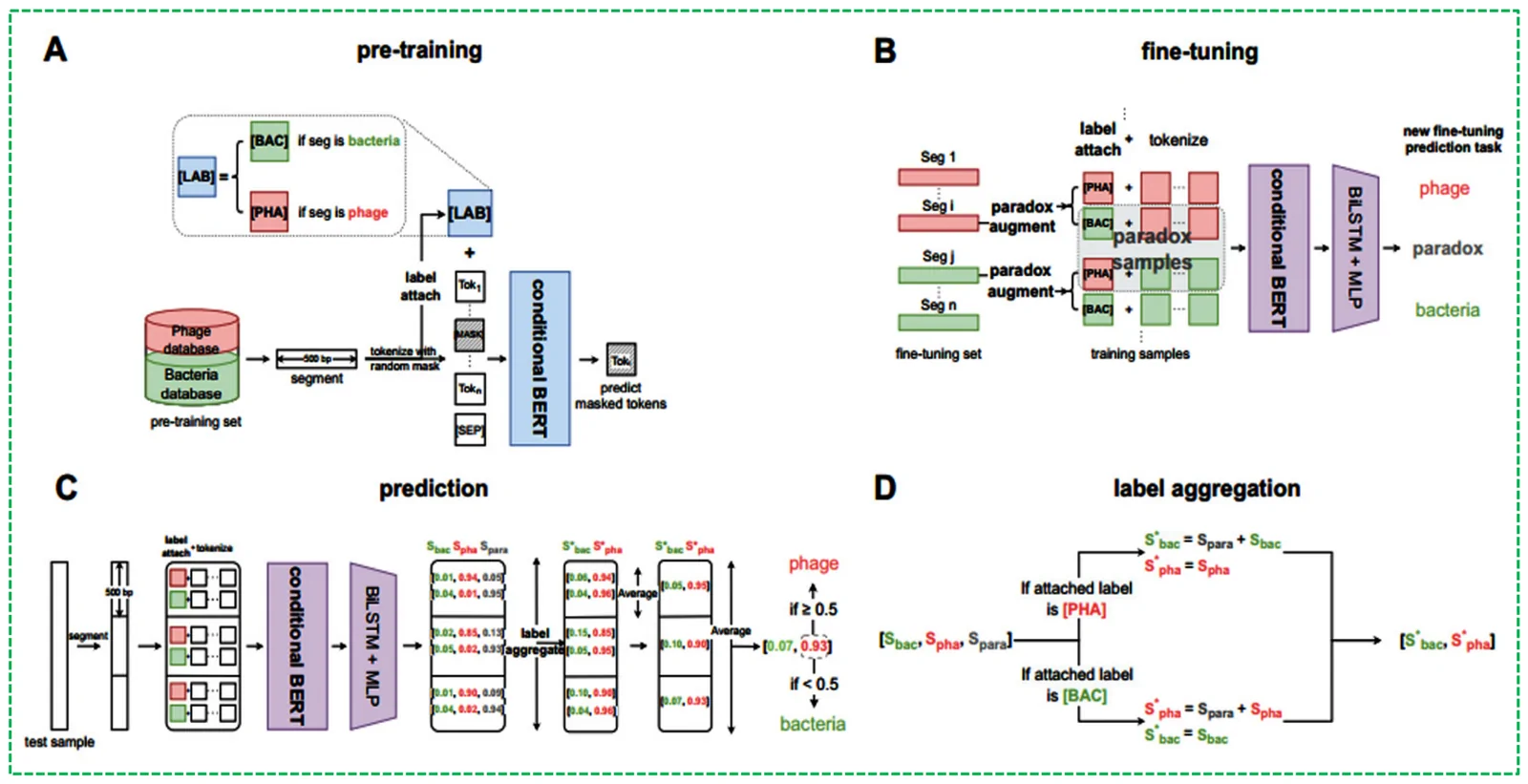
Workflow of PharaCon. (**A**) Conditional BERT is pre-trained on tokenized [BAC]/[PHA]-tagged data to learn label-specific contextual representations via masked language modeling. (**B**) Sequences are dual-tokenized with mismatched labels to construct paradox samples for fine-tuning, enabling the model to distinguish valid labels and paradox signals. (**C**) Metagenomic sequences are split into 500 bp fragments tagged with [BAC] and [PHA]. Label aggregation transforms three label scores into bacteria/phage/paradox categorical scores, which are converted to binary fractions and averaged across fragments for final prediction. (**D**) Label aggregation rules: combine bacteria and paradox scores for the [PHA] tag; combine phage and paradox scores for the [BAC] tag [48].

Utilizing machine learning to optimize bacteriophage screening is emerging as a key driver of future medical advances. Recent data-driven AI advancements have enabled high-throughput, precise, and predictable phage screening and design, accelerating the translation from environmental samples to clinical applications [38,39]. Early deep learning models, trained on phage genomic features and host information, could predict potential hosts with high accuracy [40,41]. One study developed a computational framework that integrates sequence and network information to predict phage–bacterium interactions [42]. Another demonstrated that algorithms such as SVM could effectively infer host range using only phage genomic sequences [43]. These early studies laid important groundwork for the more complex machine learning models that followed. As algorithmic techniques evolved, ensemble learning methods–including random forests and gradient boosting trees–were gradually introduced into phage screening, markedly improving prediction stability and accuracy [44,45]. Recent breakthroughs in deep learning have revolutionized phage screening. Machine learning models can efficiently identify phage sequences within massive metagenomic data from complex environments [9,46]. More critically, the Transformer architecture from natural language processing has been adapted to biological sequence analysis. Self-attention-based models such as PhageBERT can directly learn deep semantic features of phage genomic sequences without relying on predefined feature engineering, thereby uncovering sequence patterns and host association rules that traditional methods struggle to capture [47]. However, these models have some shortcomings. Specifically, most of the models are usually pretrained with self-supervised tasks. However, these tasks overlook label variance within the pretrained data, leading to significant deviations in phage recognition. The pre-training of mixed bacterial and phage data may lead to information bias because of imbalance between bacterial and phage samples. Therefore, Bai et al. introduced PharaCon, a novel conditional BERT framework, which treats label classes as special tokens during the pre-training process [48]. Specifically, during tokenization, the BERT model directly attaches labels, introducing label constraints into the model’s input (Figure 3). In addition, a new fine-tuning scheme has been introduced that allows conditional BERT to classify data effectively. The framework allows the BERT model to obtain labels, extract specific context representations from mixed sequence data in the pre-training phase, and use conditional BERT as a classifier in the fine-tuning phase. PharaCon was evaluated against several existing methods on simulated sequence and real metagenomic contig datasets. The findings show that PharaCon is a highly effective and efficient tool in phage recognition.

In real-world applications, the value of machine learning for optimizing phage screening is becoming increasingly evident. To combat multidrug-resistant bacterial infections, one research team developed an end-to-end phage screening platform that integrates bacterial phenotype data, phage lytic spectra, and individual patient characteristics into a unified modeling framework [49]. Within hours, this system can accurately match the most promising therapeutic candidate from a library of thousands of phages, compressing a traditional experimental screening process that takes weeks into an extremely short timeframe. Moreover, the introduction of active learning strategies endows the model with continuous improvement capabilities. Through a human-in-the-loop mechanism, the system can prioritize phage–bacterium pairs that offer the greatest information gain for experimental validation, maximizing screening efficiency under limited experimental budgets and creating a virtuous cycle between data-driven prediction and bench validation [50]. In summary, this intelligent phage screening system not only drastically shortens the discovery cycle of candidate phages but also achieves a dual leap in screening efficiency and model performance through active learning and human-AI collaboration. This offers an efficient and scalable technical paradigm for the clinical translation of precision phage therapy.

## 5. Application of Deep Learning in Bacteriophage Classification

Phage classification is challenging because of their high sequence diversity, lack of universal marker genes, and frequent recombination events [51]. Although multiple revisions by the International Committee on Taxonomy of Viruses (ICTV) have helped refine phage taxonomy, a systematic and efficient classification workflow is still lacking. Deep learning offers a way forward. Neural network-based models can automatically learn hierarchical feature representations from large-scale sequence data, moving beyond the need for manual feature engineering. These models have already shown outstanding performance in tasks such as protein structure prediction and gene function annotation [47,52]. In the specific area of phage classification, deep learning is now advancing the field from multiple angles, including sequence-based classification, host prediction, and lifestyle discrimination [53,54,55].

Traditional phage classification methods, including homology search based on BLAST (BLAST+ 2.17.0) and shallow machine learning algorithms like random forest, rely heavily on manual feature design of protein sequences, such as amino acid composition, dipeptide frequency, or physicochemical properties of pseudo-amino acids. These methods fail to capture long-range context dependencies in sequences and are ineffective in dealing with ‘ viral dark matter’, which is abundant in metagenomic data, in which sequences share no detectable homology with known phages [56]. In contrast, AI-driven approaches integrate sequence-derived embeddings with genomic context information to predict low-homology proteins and reconstruct candidate defense systems at scale, thereby uncovering novel systems that traditional methods cannot reach [57]. As another example, the VirHost Hunter framework uses a pretrained protein language model to extract embedding representations of phage tail fiber proteins and lysins. Even when sequence similarity is extremely low, this approach captures functional homology, effectively doubling the number of host assignments for gut phages [58].

Phage virion proteins (PVPs) are structural components of the phage capsid and tail that are crucial for host recognition and infection [59]. In recent years, deep learning-based protein classification models have markedly improved the performance of PVP identification. The ProtPhage framework introduced the ProtT5 protein language model together with an asymmetric loss function, significantly boosting prediction performance for minority classes such as “minor capsid” proteins [60]. At the same time, as a reconfigurable machine learning framework, PhageScanner integrates deep learning models, including LSTMs, enabling simultaneous PVP prediction and toxin protein identification based on genomic and metagenomic data [61]. On a related note, models like DefensePredictor leverage the ESM2 protein language model to mine bacterial defense systems at scale [62], revealing, from the opposite perspective, the complex backdrop that phage classification must contend with.

These advances show that pretrained large language models, through self-supervised learning on massive protein sequence datasets, have acquired deep linguistic rules governing protein evolution, structure, and function. As a result, they can provide an exceptionally powerful feature foundation for downstream classification tasks. Amid the ongoing convergence of AI and phage research, the application of deep learning to phage classification is becoming a critical element in the next chapter of future medicine. The accurate classification of phages is crucial for gaining a deeper understanding of their characteristics and developing phage therapies; deep learning technologies, with their powerful data analysis and pattern recognition capabilities, have brought about revolutionary breakthroughs in phage classification.

## 6. The Role of AI in Personalized Customization of Phage Therapy

AI plays a vital role in the personalized customization of phage therapy. Pirnay et al. Conducted a retrospective study of 100 personalized phage treatment cases, demonstrating that the use of customized phage selection methods for specific bacterial infections in individual patients, and the selection of personalized methods, has a significant therapeutic effect in clinical treatment [63]. Moreover, AI methods can integrate patient-specific data, bacterial characteristics, and phage properties to predict treatment outcomes and optimize phage or phage-antibiotic combinations, thereby enabling more personalized phage therapy [64]. AI can also predict bacterial resistance to phages, helping clinicians identify the best phage candidates for individualized treatment in real-world settings. By combining machine learning algorithms, researchers can rapidly pinpoint optimal phages, ensuring that patients receive the most effective therapy for their particular infection [65,66,67]. In addition, a new antibody preparation method has recently been developed that uses an AI-assisted phage display approach to generate novel variants of existing antibodies [68]. These new antibody variants have immense potential for applications in research, diagnostics, and personalized treatment.

Despite growing interest in phage therapy, several key issues continue to limit its clinical application. Personalized phage therapy remains expensive and logistically complex, particularly when phages must be isolated, characterized, and manufactured in real time [69]. The integration of AI is fundamentally reshaping this challenge. By combining genomic data from patient bacterial isolates with large-scale phage databases, machine learning algorithms can screen and match the most suitable phage candidates within hours [70]. More specifically, convolutional neural networks have been used to analyze the three-dimensional structural features of bacterial surface receptors and predict their binding affinity to phage tail fiber proteins. More importantly, deep learning models can integrate various dimensions of information such as the immune status of the diseased animals or patients, the micro-environment parameters at the infection site, and the history of previous antibiotic use, thereby constructing personalized phage mixture formulas. For example, in chronic Pseudomonas aeruginosa infections commonly seen in cystic fibrosis patients, reinforcement learning algorithms simulate the dynamic trade-offs among biofilm penetration, lysis kinetics, and resistance evasion for different phage combinations, ultimately outputting an optimal sequence of treatment regimens. Meanwhile, natural language processing is accelerating the integration of global phage banks, allowing clinicians to perform real-time searches for matched, characterized phage resources and substantially reducing the time and cost associated with de novo phage isolation. This data-driven personalized paradigm not only enhances therapeutic precision but also lays the technical groundwork for moving phage therapy from experimental intervention toward standardized medical practice.

## 7. Predicting Phage-Host Interactions (PHIs) Through AI

In the interdisciplinary field of AI and phage research, AI-based prediction of PHIs has emerged as a frontier in medical studies. Table 2 summarizes the mainstream AI-driven PHI prediction models developed over the past five years, illustrating a clear trend toward increasing technical maturity and methodological diversity in this area. For host prediction, models such as VirHostMatcher-Net improve the accuracy of PHI by integrating network frameworks with CRISPR sequence analysis [71]. In contrast, HostG offers deeper insights into the mechanisms of PHI by using semi-supervised learning and knowledge graph techniques to extend the prediction of novel viral hosts [72]. Additionally, the GSPHI platform uses deep learning to extract high-level features from bacterial receptor proteins and phage tail proteins; these features are then combined with a PHI matrix to accurately predict potential infectivity relationships [73].

**Figure 4:**
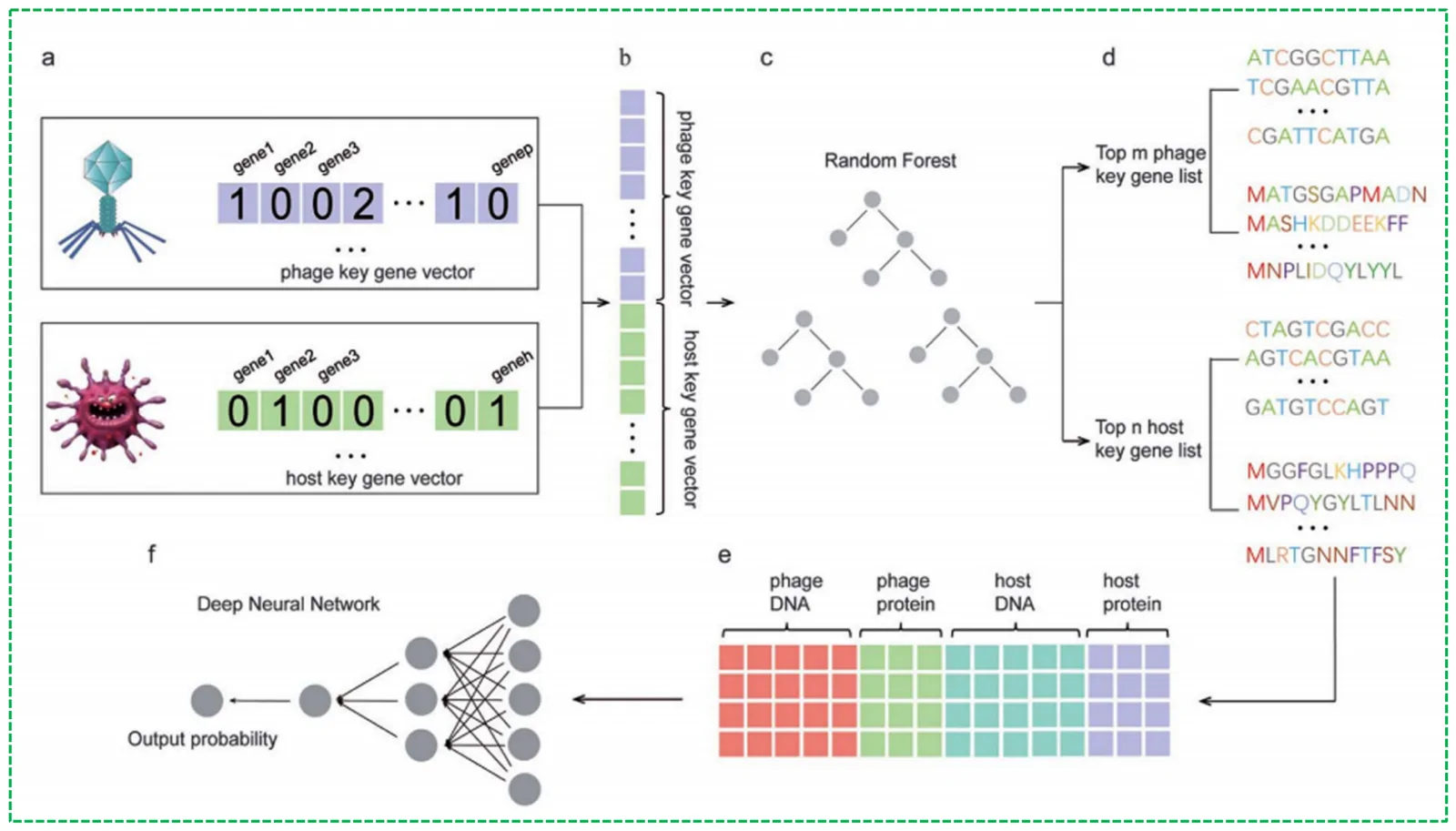
Overview of the DeepPBI-KG workflow. Knowledge graph and GO term annotations are used to screen key genes and construct feature vectors. The model adopts a five-layer deep neural network as the main prediction module, followed by a random forest re-classification step to predict phage–host interactions. (**a**) Feature vectors for key genes of phages and hosts were constructed; (**b**) Integrate the feature vectors of key genes from the host and the bacteriophage to generate a composite feature, where each sample represents a combined vector for a bacteriophage-bacteria pair; (**c**) Combine the positive and negative samples, input them into the radiofrequency (RF) model for reclassification, and calculate the features for each key gene; (**d**) Based on the preset threshold, identify genes with high scores in both the phage and the host bacterium, and extract the DNA and protein sequences corresponding to these high-scoring genes; (**e**) Compute the DNA-protein features corresponding to the top-ranked genes, and concatenate these features into a high-dimensional vector with dimensions “6 × 223 × 2” to serve as the final input features for the model; (**f**) Use a deep neural network (DNN) model with five hidden layers to predict interaction probabilities [75].

Here we focus on the currently representative DeepPBI-KG model. This model integrates four key types of nodes—phage, host, protein, and gene function—gathered from multiple databases and computational predictions. DeepPBI-KG leverages GO annotations and knowledge graph information to screen key genes and build feature vectors for phage and host bacteria. The core prediction pipeline adopts a five-layer deep neural network, followed by a random forest re-classification module to complete phage–host interaction prediction. For instance, a phage node can influence its tail fiber protein node via an encoding relationship; that protein node may then link to a “cell wall degradation” Gene Ontology (GO) term through a functional association, and this GO term can subsequently connect to a bacterial node via other proteins that share that function. Through this process, the model learns deep vector representations of nodes that embed substantial biological logic (Figure 4). In terms of predictive performance, this model substantially outperforms traditional sequence similarity-based methods. Its innovation lies in breaking through the limitations of single-data-type approaches by effectively integrating multi-dimensional associations from heterogeneous biological networks, thereby enabling the identification of novel phage–host pairs that show low sequence similarity but have potential functional interactions. This capability is particularly valuable for expanding the pool of candidates for phage therapy, because many therapeutically promising phages may have host ranges that are difficult to determine by conventional means—either due to incomplete genomic annotations or rapid sequence evolution.

At the level of translational application, AI-driven PHI prediction is increasingly being integrated into clinical decision support systems for phage therapy. In a personalized phage therapy pilot project approved by the U.S. FDA in 2023 [74], the PHI prediction module served as a core component, capable of delivering an optimal phage combination recommendation within four hours of receiving the patient’s bacterial isolate genome data. This reduces the turnaround time from weeks to days compared with traditional empirical screening approaches. This efficiency is especially critical when treating life-threatening infections caused by multidrug-resistant bacteria. Concurrently, the European Phage Bank network is working to establish standardized guidelines for interpreting PHI prediction results. By harmonizing the clinical data across different AI models, they aim to transition this field from a research tool to a regulatory-approved diagnostic aid [63]. This marks an important turning point, as AI-based PHI prediction begins its transition from laboratory research to clinical application.

**Table 2:** Main PHI Prediction Models Based on AI in the Past Five Years.

Model Name	Core Technology and Characteristic	Feature	Primary Data Format(s)/Key Limitation(s)	Reference
PHERI	A model based on sequence features and machine learning	It can identify and highlight the important protein sequences selected for the host.	Format: Protein amino acid sequences (FASTA). Limitation: Limited generalizability to novel/unseen host genera/species, as it relies heavily on known host-specific sequence markers.	[76]
HostG		It supports multi-classification and has high accuracy at the bacterial genus and species level.	Format: Whole-genome DNA sequences or k-mer frequency vectors. Limitation: Requires relatively complete genome assemblies; prediction accuracy drops significantly for fragmented short reads or draft contigs.	[72]
VirHostMatcher-Net		It performs well in species-level and genus-level prediction, especially for short sequences.	Format: Short DNA/protein sequences (e.g., contigs, scaffolds) in FASTA/Q. Limitation: Predictive features may become unstable for long genomes or phages with frequent recombination events.	[71]
DeepHost	Sequence model based on deep learning	It can capture functional domains and conserved motifs in sequences and is suitable for high-precision species-level prediction.	Format: Nucleotide or amino acid sequences (FASTA). Limitation: Requires large-scale, high-quality labeled training data; high computational resource consumption; poor interpretability for diagnostic analysis.	[17]
SCORPION		It can be used to predict phage surface proteins.	Format: Phage surface protein sequences (FASTA). Limitation: Narrow scope—exclusively designed for surface protein identification and cannot predict other types of phage-host interactions (e.g., cytoplasmic or regulatory proteins).	[77]
DeepPBI-KG		It is through key genes and proteins to achieve phage-bacterial interaction prediction.	Format: Gene/protein sequences combined with knowledge graph entity-relation triples. Limitation: Suffers from data sparsity for newly discovered or unannotated genes/proteins, as KG coverage depends on existing databases (e.g., GO, KEGG).	[75]
CM-PHI	Model based on graph neural network and network inference	Better accuracy and robustness	Format: Protein–protein interaction (PPI) networks (adjacency matrix or edge list) with node features. Limitation: PPI network construction relies heavily on experimentally validated or predicted interaction data; performance drops sharply for systems with sparse or no interaction records.	[78]
IK-BRNet		The model does not depend on a single data source but integrates multiple types of data.	Format: Multi-source heterogeneous data (sequences, networks, biochemical properties, the literature-mined features) integrated as tensors or feature matrices. Limitation: Integration pipeline is complex; noise and biases from individual data sources accumulate, affecting overall robustness.	[79]
MetaPHinder		Optimized for metagenomic data, it is suitable for host prediction in complex microbial communities.	Format: Metagenomic shotgun reads, assembled contigs, or MAGs (bins). Limitation: Limited resolution for distinguishing low-abundance or highly similar strains; computationally expensive as dataset size grows.	[80]
PHPGAT	Based on multi-modal fusion and a pre-trained large model	The “cold start” problem can be solved in a targeted manner.	Format: Multi-modal inputs (joint embeddings of sequences, predicted protein structures, and functional annotation labels). Limitation: Pre-trained models may carry inherent biases toward frequent training patterns; still requires target-domain fine-tuning with minimal labeled data; high inference cost due to large parameter size.	[81]
HostNet		It supports multi-level prediction from molecular mechanism to ecological scale, which is suitable for engineering phage design.	Format: Multi-scale data (molecular sequences, biological interaction networks, host physiological/ecological metadata, environmental parameters). Limitation: Requires simultaneous acquisition of diverse, large-scale datasets; ecological metadata are often difficult to standardize and obtain, restricting broad practical applicability.	[82]

Note: Reported performance metrics are obtained under specific benchmark conditions. Model behavior can be strongly influenced by dataset composition, taxon sampling bias, and class imbalance. High in silico accuracy does not guarantee successful wet-lab validation. Computational resource requirements vary substantially across different algorithm categories.

Recently, several studies have successfully used AI to predict phage infections at the strain level directly from host genome sequences, achieving strain-level predictions for the genera *Klebsiella* and *Escherichia* [83,84]. These works reflect the current state of the art. This framework builds a PHI knowledge graph using graph convolutional networks (GCNs) and successfully achieves zero-shot transfer to data-scarce genera such as *Vibrio* and *Alteromonas*. This advance suggests that future phage classification systems will no longer be satisfied with simple species labels; instead, they will evolve toward the more complex and practically valuable goal of dynamically predicting the host range of phages at the strain level.

AI also plays an important role in analyzing and predicting the dynamic interplay between phages and bacteria. Over the course of long-term co-evolution, bacteria and phages continuously adapt to and counteract each other, with bacteria gradually developing resistance to phages [85,86,87]. By simulating the PHI process with AI, researchers can predict the likely trajectory of phage resistance. For example, using agent-based modeling, they can simulate population dynamics between phages and bacteria under different environmental conditions and analyze the factors that influence the emergence of phage resistance. Such studies have found that under certain specific environmental conditions, the probability of bacteria developing phage resistance increases markedly [88]. These predictions help in developing proactive strategies, such as adjusting the phage administration regimen, to ensure the effectiveness of phage therapy. AI serves as a powerful tool for responding swiftly to changes in PHI.

## 8. Application of AI in Simulating the Interaction Between Phage and Bacteria

It is well known that the first step in bacteriophage infection of bacteria is adsorption, which is mediated by the specific recognition between the phage tail protein and bacterial surface receptors [89,90]. Accurately modeling the adsorption process is therefore crucial for understanding phage host range. To address this, Ma and colleagues proposed the PBIP deep learning framework, which constructs a heterogeneous microbial network centered on adsorption-related proteins. The framework uses a multi-hop attention graph neural network (MHAGNN) to capture topological features, while a bidirectional gated convolutional neural network (GCNN) encodes sequence semantic information, enabling precise prediction of PHI [91]. No single aggregated accuracy value was provided in the original publication; this model was further combined with AlphaFold 3 for molecular docking analysis, which validated the reliability and interpretability of the predictions. These works indicate that AI has been able to effectively decode and recognize the infection initiation signal from the genome sequence. Separately, CoMPHI (Composite Model for Phage Host Interaction) introduces a composite framework that integrates alignment methods, generating multi-feature encodings from nucleotide and protein sequences (Comparative summaries for these phage-host interaction prediction models are available in Table 2). It combines alignment scores at three levels—phage–phage, phage–host, and host–host—to make predictions. As originally reported, the CoMPHI composite framework obtains prediction accuracies ranging from 92.3% to 95.1% under its benchmark evaluation setup [92].

Accurately predicting the host range of phages at the strain level is a central prerequisite for the clinical application of phage therapy. In terms of algorithm fusion, the MoEPH model adopts the Mixture-of-Experts architecture, combines Transformer-based protein embeddings such as ProtBERT and ProT5 with domain-specific statistical descriptors, and realizes adaptive prediction through the gated fusion mechanism. On its balanced test dataset, the MoEPH model yields an accuracy of 99.6%; meanwhile, it achieves a 31% relative improvement in prediction performance compared with baseline models on the unbalanced dataset [93]. The PhageMind framework proposed by Shen et al. innovatively incorporates meta-learning strategies, demonstrating strong generalization capabilities in cross-genus validation and enabling rapid adaptation to prediction tasks for new bacterial genera with only a small number of known interactions [94]. For Clostridioides difficile, the PHISDetector tool integrates multiple computational signals and reaches 85.6% accuracy for predicting phages infecting *Clostridioides difficile* on its test set, according to the original study [95]. Finally, Malajczuk et al. conducted a systematic review of the aforementioned strain-level prediction methods, noting that data sparsity, reliance on experimental labels, and poorly designed benchmarks are the primary challenges currently faced [41].

AI is driving a paradigm shift in phage therapy as it transitions from simulating interactions to guiding clinical treatment decisions. Rahimian proposed a fully automated phage therapy pipeline for intelligent hospitals in Medical Hypotheses. It involves integration of physics-informed neural networks, IoT robotic systems, and real-time metagenomic feedback to achieve closed-loop optimization of patient-specific phage screening and personalized dosing [96]. Meanwhile, the GE-PHI framework combines knowledge graph embeddings with the ESM-2 protein language model to extract evolutionary information from phage tail proteins and host receptor binding proteins, achieving a cross-validation AUC of 0.9453 in computational simulations. The MVPHI model extracts microbial features from three perspectives—statistical, textual, and topological—providing a multi-view analytical framework for phage screening and bacterial community studies [97].

In summary, the application of AI to simulating PHIs has brought revolutionary changes to phage research and phage therapy. It not only deepens our understanding of the relationship between phages and bacteria but also offers more effective strategies and tools for future medicine in combating bacterial infections, representing an important innovative application of AI in the field of phage research.

## 9. Using AI to Predict the Development of Phage Resistance

Bacteria have evolved diverse resistance mechanisms under the selective pressure of bacteriophages. This poses a central and significant challenge for the clinical translation of phage therapy. Traditional phage screening strategies, which rely on in vitro experiments, are time-consuming and labor-intensive, and often fail to predict the trajectory of bacterial resistance evolution [98]. In recent years, artificial intelligence and machine learning have offered new perspectives and tools for predicting phage–bacterium interactions and resistance development at the genomic level [99]. This section reviews the potential, technical advances, and challenges in applying AI to predict phage resistance evolution.

First, supervised learning models based on high-throughput sequencing data have strong capabilities in phenotypic prediction. Lucia-Sanz and colleagues trained a machine learning framework on genomic data to predict the infectivity of a phage against specific bacterial strains and the efficiency of infection. Their model successfully predicted 86% of potential interactions, reduced the relative error of infection efficiency by 40%, and identified mutations in several genes with unknown functions [100]. This provides new insights into the molecular basis of bacterial resistance. However, bacterial resistance is a dynamic evolutionary process [101,102], and static interaction prediction alone is insufficient for clinical needs. A combined framework integrating generative models with predictors offers a more effective solution. Ataee et al. proposed a deep learning architecture in which a generator modifies phage genomes with high accuracy to expand host range. Their data demonstrate that AI can not only predict the host range of existing phages but also guide phage engineering to circumvent bacterial resistance [103].

In the context of phage resistance prediction, DeWeirdt et al. developed DefensePredictor using the protein language model ESM2. This tool identifies anti-phage defense proteins and reveals that the diversity of bacterial immune systems is far greater than previously recognized, offering a means to assess bacterial resistance potential [63]. That said, the model would benefit from integrating genomic context and regulatory network information to improve its predictive accuracy.

Taken together, the above studies suggest that AI is moving beyond static interaction predictions toward dynamic modeling of resistance evolution and active engineering interventions in the phage field. Nevertheless, several key challenges remain. For one, most current models rely on laboratory co-evolution data, whereas clinical environments are far more complex than laboratory systems [104]. Maintaining model generalizability with sparse and noisy real-world clinical samples is still a pressing issue. Second, bacterial resistance mechanisms include not only adaptive immune systems like CRISPR-Cas but also receptor loss, restriction-modification systems, abortive infection, and other strategies [105]. While current AI models perform reasonably well in predicting receptor-level resistance, they lack effective ways to model complex resistance mechanisms that involve signaling pathway regulation and metabolic network reprogramming. For example, Pons et al. noted that even under CRISPR-Cas immune protection, phage infection can still induce growth arrest, filamentation, and the SOS response in the host. This suggests that the resistance phenotypes involve complex mechanisms and cannot be explained by genomic mutations alone [106].

In summary, AI has shown substantial potential in predicting phage resistance development. From deciphering the genetic basis of resistance to actively engineering phages that circumvent resistance, and from predicting the distribution of bacterial defense systems to reshaping the research paradigm of phage therapy, AI is making its mark across multiple fronts. Nevertheless, a considerable gap remains between laboratory models and clinical application. Future research will need to make sustained efforts in multimodal data integration, dynamic evolutionary modeling, improving model interpretability, and clinical validation before AI-driven precision phage therapy can become a reality.

## 10. Application of AI in Phageomics Data Mining

In recent years, the widespread adoption of high-throughput sequencing has accelerated the discovery of large-scale phage genomes considerably. However, a growing gap has emerged between the rapid accumulation of phage genomic data and the ability to functionally mine it. It is estimated that roughly 65% of phage protein sequences lack reliable biological annotation through traditional homology-based methods. This creates a substantial amount of genomic “dark matter” in phage biology research [42]. Against this backdrop, AI is fundamentally reshaping the data analysis paradigm in phage genomics, offering a new technical path to systematically address this data mining bottleneck [47]. This section reviews cutting-edge applications of AI in phage genomics data mining across three core areas: functional annotation and identification, prediction of PHI, and lifestyle classification along with genome design.

Functional annotation and sequence identification represent the most direct applications of AI in phageomics. Traditional methods rely heavily on sequence homology searches. However, many phage proteins lack known homologs, leading to annotation coverage far lower than that achieved in bacterial systems. To address this challenge, Guan et al. proposed the GOPhage method, which infers the functions of uncharacterized proteins by integrating colocalization information of adjacent genes in phage genomes [107]. Using GOPhage, the study successfully identified 688 potential holins, which show high structural conservation with known holins. This demonstrated the method’s potential to expand our understanding of newly discovered phages. Meanwhile, deep learning-based identification tools now enable efficient and rapid sequence identification, allowing for the discrimination of bacterial and phage sequences without sequence alignment, and maintaining stable performance even under conditions of extremely low sequence similarity [9,108]. Furthermore, a new generation of deep learning frameworks is extending the analytical scope from individual genes to the whole-genome scale, offering unprecedented efficiency for phage mining in large-scale metagenomic data.

Accurate PHI prediction serves as a cornerstone for phage therapy and microbial ecology research, and AI models have made notable progress in this area. Shang et al. systematically benchmarked 27 host prediction tools and found that no single tool universally outperformed others. Rather, their performance depended strongly on the application scenario and data characteristics, with a key tradeoff between predictive accuracy and computational cost [109]. To overcome this limitation, several deep learning frameworks have been proposed. From a metagenomic perspective, the PHILM framework learns PHI directly from taxonomic profiles; it identified 90% more genus-level interactions in 7016 healthy human fecal samples than traditional assembly-based methods [93].

Machine learning is now pushing the classification of phage life cycles from fragmented prediction toward whole-genome design. A phage’s life cycle (lytic vs. temperate/lysogenic) determines its antibacterial activity and ecological behavior, and accurate classification is critical for phage therapy and ecological studies. At the forefront, the genomic foundation model Evo—trained on over two million phage genomes—was fine-tuned on Microviridae sequences through supervised learning, generating 302 candidate genomes. Experimental validation of some of these designed phages showed that they can infect *Escherichia coli* (including drug-resistant strains), with infection efficiencies surpassing those of their natural counterparts [110,111,112,113]. This work represents the first instance of generative whole-genome design of phages validated by experiments, marking a paradigm shift in phageomics from passive discovery to active design.

## 11. Ethical Issues of AI in Phage Research

As AI advances rapidly in phage research and its capabilities continue to break new ground, a number of ethical challenges have also emerged, including biosafety risks, data bias, attribution of algorithmic responsibility, and a lack of global governance frameworks. The dual-use nature of AI-driven phage research has sparked controversy: the same technology that can support clinical therapy could also be misused for harmful purposes. The phages designed by the Evo model outperform natural viruses in bacterial killing and overcoming drug resistance—a technical achievement that could potentially be exploited by non-state actors or hostile nations. The dual-use dilemma in phage AI research is even more complex than that of traditional pathogen genetic modification, because AI-based design requires only genomic sequence information, making regulation and traceability difficult while also raising the risk of “emergent” harms. In response, academic voices have called for stronger safeguards. In July 2025, the AIxBio Global Forum issued the Statement on Biosafety Risks at the Intersection of AI and the Life Sciences, first published on the NTI official website [NTI 2025]. Jointly signed by more than 35 AI developers and international security experts, the statement urges all parties to establish technical guardrails and governance mechanisms [NTI News 2025].

When an AI model makes an error in functional prediction during phage research, assigning responsibility becomes complicated, leaving a “responsibility vacuum” within traditional ethical frameworks. If an AI-driven host prediction model recommends an ineffective or even harmful phage combination without being able to explain its reasoning, clinical prescribers face a genuine dilemma. They must strike a workable balance between relying on AI outputs and exercising their own professional judgment. Researchers have warned about the risks of applying AI in genomics: when humans can no longer monitor model outputs, some life-or-death decisions are effectively delegated to algorithms—a risk that becomes particularly acute in the clinical setting of phage therapy. Currently, global regulation of phage therapy remains highly fragmented. In most parts of Europe and the United States, it is still regarded as an “experimental” treatment, with no unified approval pathway or pharmaceutical quality standards. Large-scale production and clinical application face three major hurdles, and the deeper integration of AI only makes the regulatory landscape more challenging.

In response to these governance gaps, the academic community has proposed multi-level regulatory frameworks and called for a joint governance system. One comprehensive literature review outlines governance options and their applicability boundaries across four dimensions, arguing that governance intensity should be dynamically adjusted according to the risk scenario. The application of AI in phageomics holds great promise for reshaping precision antimicrobial strategies, but it must be guided by ethical reflection and institutional design. In 2025, 25 axioms on the governance of AI-life science integration were released, echoing the Asilomar Conference fifty years ago: technological leaps and safety safeguards should be co-designed from the earliest stages of innovation. Looking ahead, AI research in phageology should focus on four priority directions: establishing global, standardized biosecurity protocols; promoting evenly distributed global sampling of public phage databases; developing explainable AI methods; and setting up interdisciplinary ethics review boards. The ethical issues surrounding AI in phage research constitute a “necessary steering system” that ensures the technology remains robust and sustainable in serving human health and environmental well-being.

## 12. Limitations and Challenges of AI in Phage Research

As the convergence of AI and phage research opens a new chapter for future medicine, it is equally important to acknowledge the current limitations and challenges. Despite the promising potential of AI across areas such as phage identification, many unknowns remain. First, at the data level, AI relies on large volumes of high-quality data for model training. Yet obtaining such data in phage research is difficult; the number of sequenced phage genomes represents only a tiny fraction of what exists in nature. This introduces data bias in model training. For instance, when machine learning is used to prioritize phages for therapeutic screening, it may overlook those with genuine clinical potential. Second, from an ethical standpoint, the application of AI in phage research raises concerns. As Stephen Hawking once noted, the rise of powerful AI could be either the best or the worst thing to happen to humanity. If the deep mining and use of biological information by AI are not properly governed, biosafety risks may arise—for example, custom-designed phage therapies could be deliberately misused, threatening human health and ecological balance. Finally, AI faces considerable challenges in predicting PHI and the evolution of phage resistance. The interplay between phages and bacteria is highly dynamic and complex, shaped by multiple factors. Current AI models still struggle to capture this complexity fully, which limits the accuracy of their predictions and introduces uncertainties for real-world phage therapy applications.

In conclusion, in the face of these limitations and challenges, we should proceed with caution in applying AI in phage research. On one hand, we need to improve the data collection and management system to enhance data quality; on the other hand, we should strengthen the evaluation and improvement of AI models to increase their accuracy and applicability. At the same time, we should establish sound ethical norms and regulatory mechanisms to ensure that AI applications are in line with human interests and moral standards.

## 13. Summary

This review summarizes the innovative applications of AI in phage research and their far-reaching implications for future medicine. In the face of the global antibiotic resistance crisis, phage therapy has re-emerged as a precision antibacterial strategy. However, traditional research methods have long been constrained by low screening efficiency and difficulties in predicting host range. Through machine learning, deep learning, and natural language processing, AI has achieved breakthroughs in phage identification, genomic functional annotation, host interaction prediction, and whole-genome design—pushing the field from passive discovery toward active design.

This review also discusses the ethical challenges and technical limitations of AI in this context. At the data level, genomic sampling bias remains a concern. At the model level, accurate prediction of dynamic PHI remains elusive. Ethically, issues include dual-use biosafety risks, ambiguous attribution of algorithmic responsibility, and the lack of global regulatory frameworks. To address these problems, this review proposes governance pathways that include the establishment of standardized biosecurity protocols, promotion of globally representative sampling in phage databases, development of explainable AI methods, and the creation of interdisciplinary ethics review mechanisms.

In summary, the deep integration of AI into phage research offers transformative tools to combat antimicrobial resistance. Nevertheless, robust and sustainable progress will depend on improvements in data quality, algorithmic transparency, and ethical guardrails. Future research should focus on multimodal data integration and closed-loop experimental validation. Through the synergistic advancement of technological innovation and institutional design, the efficient translation of phage therapy from bench to bedside can be realized, providing new safeguards for global public health security.

## Figures and Tables

**Table 1:** List of Current Main Phage AI Recognition Tools.

Tool Name	Core Technology	Advantages	Weaknesses	References
VirFinder	K-mer frequency + Logistic Regression	It has high sensitivity to new types of phages that are underrepresented in the reference database and can perform calculations very quickly.	The false positive rate is relatively high; reliance on k-mer features may miss certain biological signals.	[18]
DeepVirFinder	k-mer + CNN	The upgraded version of VirFinder based on deep learning has higher sensitivity and is particularly adept at identifying new bacteriophages.	There may be a considerable number of false positives; it is more sensitive to contamination by eukaryotes.	[19]
VirSorter2	Probability model + Random Forest	The false positive rate is low; it is highly robust against true contamination.	The ability to distinguish plasmid sequences is limited.	[20]
DeepPL	Natural Language Processing (NLP) model based on DNABERT	Focuses on predicting the life cycle of bacteriophages (lysogenic/lytic); the accuracy rate is 94.65%; still has a high accuracy rate for short sequences (<5 kb).	Focuses on lifecycle prediction rather than general sequence recognition.	[12]
DeepHost	Genome encodes CNN	No sequence alignment, fast operation speed, outstanding cross-homologous sequence prediction capability.	The dataset is highly dependent, and the classification coverage has boundaries.	[17]
PIDE	Protein large language model (ESM-2) + Gene density clustering	When predicting the boundaries of the original phage, it can better balance the recall rate and the accuracy; it can also uncover areas that other tools have not covered.	To be released in 2025, mainly targeting the detection of the original phage island, with a limited scope.	[21]
PPR-Meta	CNN	The first three-classifier capable of simultaneously identifying both phage and plasmid fragments; it performs exceptionally well in differentiating viral and microbial contigs.	The model is quite complex and has numerous parameters.	[14]

Note: Reported performance metrics are obtained under specific benchmark conditions. Model behavior can be strongly influenced by dataset composition, taxon sampling bias, and class imbalance. High in silico accuracy does not guarantee successful wet-lab validation. Computational resource requirements vary substantially across different algorithm categories.

## Data Availability

No new data were created or analyzed in this study. Data sharing is not applicable to this article.

## Abbreviations

The following abbreviations are used in this manuscript:

AI	Artificial intelligence
SVM	Support vector machine
CNN	convolutional neural network
PHI	phage-host interaction
ICTV	International Committee on Taxonomy of Viruses
PLM	protein language model
PVP	phage virion proteins
GO	gene ontology
GCN	graph convolutional network
RNN	recurrent neural network
MHAGNN	multi-hop attention graph neural network
PINNs	physics-informed neural networks
